# Overall protein structure quality assessment using hydrogen-bonding parameters

**DOI:** 10.1107/S2059798323005077

**Published:** 2023-07-11

**Authors:** Pavel V. Afonine, Oleg V. Sobolev, Nigel W. Moriarty, Thomas C. Terwilliger, Paul D. Adams

**Affiliations:** a Molecular Biophysics and Integrated Bioimaging Division, Lawrence Berkeley National Laboratory, Berkeley, CA 94720, USA; b Bioscience Division, Los Alamos National Laboratory, Mail Stop M888, Los Alamos, NM 87545, USA; c New Mexico Consortium, Los Alamos, NM 87544, USA; d Department of Bioengineering, University of California Berkeley, Berkeley, CA 94720, USA; MRC Laboratory of Molecular Biology, United Kingdom

**Keywords:** atomic model refinement, hydrogen bonds, crystallography, cryo-EM, validation

## Abstract

Atomic model refinement at low resolution is often a challenging task. This is mostly because the experimental data are not sufficiently detailed to be described by atomic models. To make refinement practical and ensure that a refined atomic model is geometrically meaningful, additional information needs to be used such as restraints on Ramachandran plot distributions or residue side-chain rotameric states. However, using Ramachandran plots or rotameric states as refinement targets diminishes the validating power of these tools. Therefore, finding additional model-validation criteria that are not used or are difficult to use as refinement goals is desirable. Hydrogen bonds are one of the important noncovalent interactions that shape and maintain protein structure. These interactions can be characterized by a specific geometry of hydrogen donor and acceptor atoms. Systematic analysis of these geometries performed for quality-filtered high-resolution models of proteins from the Protein Data Bank shows that they have a distinct and a conserved distribution. Here, it is demonstrated how this information can be used for atomic model validation.

## Abstract 2

A new protein structure validation method using hydrogen-bonding parameters is described.

## 1. Introduction

**Figure 1:**
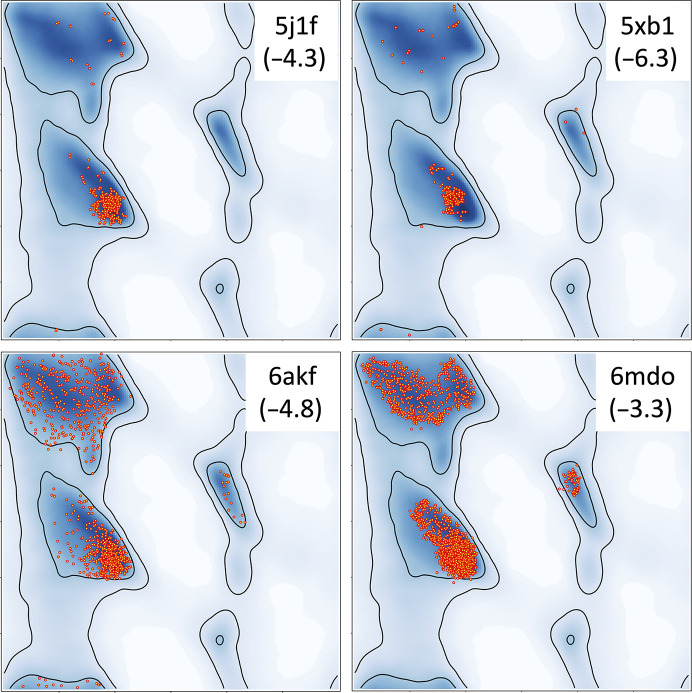
Ramachandran plots for the four models in Table 1 ▸. Rama-*Z* values are shown in parentheses. Rama-*Z* interpretation guide from Sobolev *et al.* (2020 ▸): poor, |Rama-*Z*| > 3; suspicious, 2 < |Rama-*Z*| < 3; good, |Rama-*Z*| < 2.

Validation of atomic models is an important step in structure-determination pipelines using methods such as crystallography and cryo-EM (Chen *et al.*, 2010 ▸; Richardson *et al.*, 2018 ▸; Williams *et al.*, 2018 ▸; Afonine, Klaholz *et al.*, 2018 ▸; Pintilie & Chiu, 2021 ▸). With the cryo-EM revolution (Kühlbrandt, 2014 ▸; Henderson, 2015 ▸; Nogales, 2016 ▸; Orlov *et al.*, 2017 ▸; Baldwin *et al.*, 2018 ▸), the number of structures being solved at resolutions of 3 Å and worse has constantly been increasing (see, for example, Fig. 2 in Liebschner *et al.*, 2019 ▸). Atomic model refinement at these resolutions is challenging. It requires the use of as much *a priori* information as possible to compensate for the lack of data (Schröder *et al.*, 2010 ▸; Nicholls *et al.*, 2012 ▸; Headd *et al.*, 2012 ▸; DiMaio *et al.*, 2013 ▸). This information is typically used as restraints or constraints (for a review, see Urzhumtsev & Lunin, 2019 ▸). Standard restraints are insufficient at low resolution and the use of additional restraints involving the Ramachandran plot, C^β^ deviations, residue side-chain distributions and a reference model is beneficial (Nicholls *et al.*, 2012 ▸; Headd *et al.*, 2012 ▸; Smart *et al.*, 2012 ▸; Afonine, Poon *et al.*, 2018 ▸; van Beusekom *et al.*, 2018 ▸; Casañal *et al.*, 2020 ▸). While using these extra restraints in refinement is vital to obtain chemically meaningful models, it diminishes the validating power of these tools, as they can no longer be considered as independent validators. In turn, this can lead to atomic models that satisfy all of the conventional validation criteria yet possess unrealistic geometries (Table 1 ▸ and Fig. 1 ▸). All four models in Table 1 ▸ meet or exceed *MolProbity* (http://molprobity.biochem.duke.edu/; Chen *et al.*, 2010 ▸) validation thresholds. At the same time, it is apparent that there is something very unusual about the Ramachandran plots for each of these models (Fig. 1 ▸). Residues in PDB entry 5j1f cluster around the most prominent peak in the α region in a rather circle-symmetric way. Residues in PDB entry 5xb1 systematically avoid the largest central peak in the α region and cluster near it. Furthermore, the β region is almost uniformly filled with residues in the case of PDB entry 6akf and residues in the α region follow a similar pattern to PDB entry 5j1f. Almost no residues are found in the ‘allowed but not optimal’ region, which is unlikely given the total number of residues in this model. Finally, residues in PDB entry 6mdo systematically fill the most likely areas of the β and α regions, forming sharp borders. None of these four plots can be flagged as unlikely based on favored and outlier counts, but require a trained eye for identification or the use of the Ramachandran plot *Z*-score (Rama-*Z*; Hooft *et al.*, 1997 ▸; Sobolev *et al.*, 2020 ▸), which has not yet been widely adopted in model-validation reports. Also, overall favorable validation metrics make it less likely that the researcher will look at the detailed validation reports, which include numerous plots and tables. Consequently, this increases the chance of subtle yet important model-geometry issues going unnoticed. Therefore, searching for new validation tools that are not used as refinement targets (or are difficult to use in refinement in a way to realistically describe model properties) is important.

The idea of using hydrogen-bond parameters as a validation tool for atomic models of crystal and cryo-EM structures is not new (McDonald & Thornton, 1994 ▸; Hooft *et al.*, 1996 ▸; Read *et al.*, 2011 ▸; Lawson *et al.*, 2021 ▸). Here, we introduce a new protein model-validation method that is based on the analysis of hydrogen-bond parameter distributions in available high-quality models in the Protein Data Bank (PDB; Bernstein *et al.*, 1977 ▸; Burley *et al.*, 2019 ▸). We use the examples from Table 1 ▸ and others to demonstrate the utility and uniqueness of the method. The tool has been implemented in *cctbx* (Grosse-Kunstleve *et al.*, 2002 ▸) and is also available as part of the standard validation toolset in *Phenix* (Liebschner *et al.*, 2019 ▸).

## 2. Methods

The method described here is based on an analysis of hydrogen-bond parameters extracted from high-quality atomic models of proteins available in the PDB. To perform this analysis, two entities need to be defined: the geometrical model for a hydrogen bond and criteria for selecting a high-quality set of atomic models.

**Figure 2:**
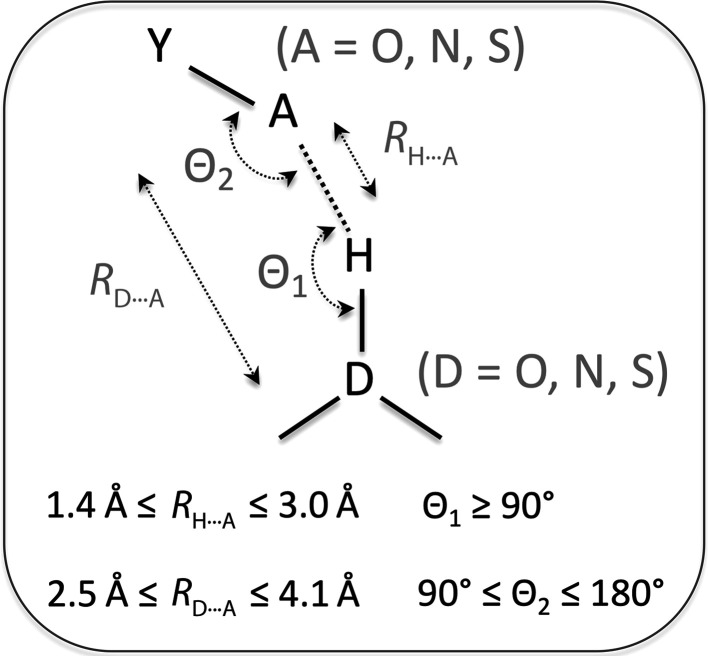
Schematic diagram to illustrate the hydrogen-bond definition used in this work. Y, A and D represent non-H atoms, H represents an H atom, solid lines represent covalent bonds, the dashed line represents a noncovalent interaction (hydrogen bond) between A and H, and double-ended arrowed straight or curvy lines represent the corresponding distances and angles.

There are several possible ways to define and parameterize hydrogen bonds (for example, Herschlag & Pinney, 2018 ▸). For the purpose of this analysis the particular choice of the parameterization used is not critical and we choose to use that shown in Fig. 2 ▸ (McDonald & Thornton, 1994 ▸).

For the selection of high-quality models, we focused on all high-resolution (1.5 Å or better) entries in the PDB obtained using crystallography and containing protein chains. Filtering by geometric quality included requirements to have less than 1% Ramachandran plot outliers and more than 95% of residues in the favored region of the plot, a *MolProbity* clashscore of less than 10, no more than 2% of residue side-chain rotamer outliers, less than 0.1% C^β^ violations and root-mean-square deviations from library values for covalent bond lengths and angles (Engh & Huber, 1991 ▸, 2001 ▸; Vagin & Murshudov, 2004 ▸; Vagin *et al.*, 2004 ▸; Moriarty *et al.*, 2016 ▸; Moriarty & Adams, 2021 ▸) of less than 0.03 Å and 3°, respectively.

To annotate hydrogen bonds, we added a new tool to *Phenix* called *phenix.hbond* that finds hydrogen bonds using the definition in Fig. 2 ▸. *Reduce* (Word *et al.*, 1999 ▸) is used as part of *phenix.hbond* to add H atoms to the model. To focus on well ordered atoms only, atoms with an ADP of greater than 30 Å^2^ and an occupancy of less then 0.9 were filtered out. The definition of hydrogen bond used here coupled with possible model-geometry imperfections may potentially allow the detection of spurious hydrogen bonds; all hydrogen bonds that satisfy the criteria stated in Fig. 2 ▸ were considered.

We conducted all analyses separately for α-helices, β-sheets and all atoms. Only hydrogen bonds between backbone atoms were considered when focusing on α-helices and β-sheets, otherwise all hydrogen bonds were used. Popular secondary-structure annotation procedures, such as *DSSP* (Kabsch & Sander, 1983 ▸), rely heavily on the geometry of hydrogen bonds, which may potentially bias our analyses. Therefore, here we used an alternative method available in *Phenix* (*phenix.find_ss_from_ca*; Terwilliger *et al.*, 2018 ▸). The method uses the mutual positions of C^α^ atoms and does not explicitly use any of the parameters of hydrogen bonds from the definition in Fig. 2 ▸.

Some low-resolution models happen to have higher resolution homologs in the PDB. This makes it possible to compare more realistic parameters extracted from high-resolution models with those derived from the low-resolution models. We used the *phenix.homology* tool in *Phenix* (Xu *et al.*, 2020 ▸) to identify these models and use them in the following analyses.

Finally, we performed a numeric experiment that illustrates (i) how an atomic model refinement using low-resolution data with insufficiently parameterized geometric restraints can lead to significant deviations of hydrogen-bond parameter values from the expected ranges and (ii) how a more appropriate (for the data resolution) choice of parameterization helps to alleviate this issue.

## 3. Results and discussion

**Figure 3:**
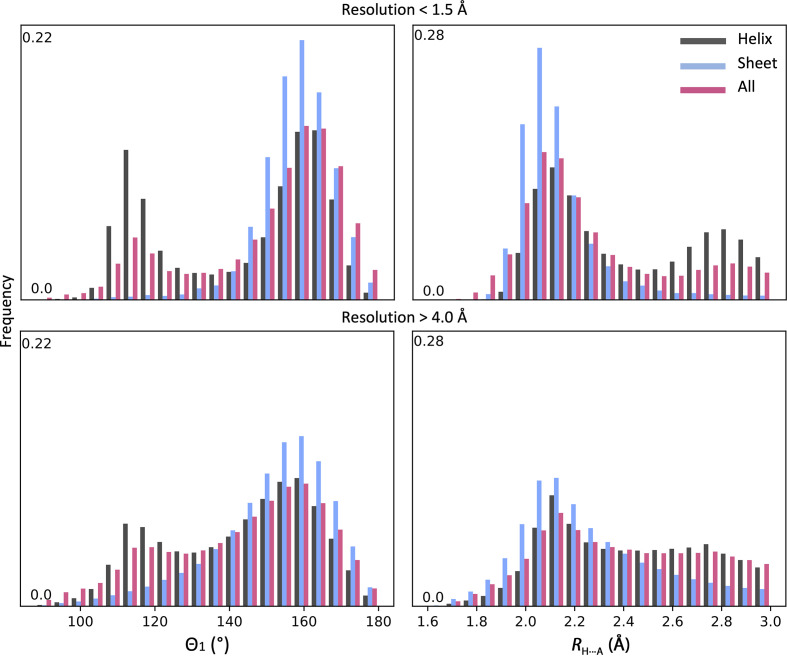
Distribution of Θ_1_ angles and *R*_H⋯A_ distances for all models in the PDB at resolutions of better than 1.5 Å (2941 models) and worse than 4.0 Å (4712 models). The number of hydrogen bonds considered in the high-resolution set are 356 797, 109 233 and 1 038 363 for α-helices, β-sheets and total, respectively. The total numbers of hydrogen bonds considered in the low-resolution set are 6 676 005, 1 191 779 and 13 480 683 for α-helices, β-sheets and total, respectively.

Fig. 3 ▸ shows the distributions of *R*_H⋯A_ distances and Θ_1_ angles for the filtered (as described in Section 2) set of high-resolution models and for all models with a reported resolution worse than 4 Å. The distributions are shown for all atoms as well as for α-helices and β-sheets separately. We make three key observations about these distributions. The distributions are skewed, vary by secondary-structure type and appear to be different for high- and low-resolution models. One of the possible reasons for this difference is the lack of information about secondary-structure geometry in the low-resolution data and the limited ability of modeling tools to account for noncovalent interactions such as hydrogen bonds.

We also observe that the peak centers for both (*R*_H⋯A_ and Θ_1_) distributions vary only slightly for high- versus low-resolution models, while the shapes of the distributions change more prominently. Fig. 3 ▸ shows the accumulated distribution for all bonds in all models selected, while it does not show how much the distribution of *R*_H⋯A_ and Θ_1_ varies from model to model, which would require the calculation and analysis of the individual distributions for each model, a tedious exercise considering the number of models. Skew and kurtosis are useful mathematical tools when it comes to the analysis of the shapes of distributions, and thus we use them in the following to characterize the distributions of *R*_H⋯A_ and Θ_1_.

**Figure 4:**
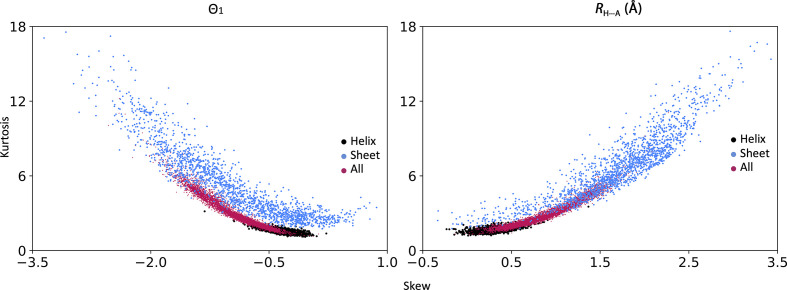
Distribution of skew versus kurtosis for Θ_1_ angles and *R*_H⋯A_ distances for high-resolution models in the PDB shown for α-helices (black), β-sheets (blue) and the whole model (magenta).

Fig. 4 ▸ shows a scatter plot of skew versus kurtosis for Θ_1_ and *R*_H⋯A_ for α-helices, β-sheets and whole models. Clearly the distributions are clustered and occupy different regions of the plot (with some overlap). It can be concluded that the distributions of Θ_1_ and *R*_H⋯A_ are rather characteristic and well conserved across protein structures and therefore can be tabulated for use as a reference. The distribution for the β-sheets is much less ordered, which is likely to be owing to the greater flexibility of β-structures. These plots define the expected range of skew and kurtosis values and suggest that values far from the clustered regions are indicative of atomic model anomalies.

**Figure 5:**
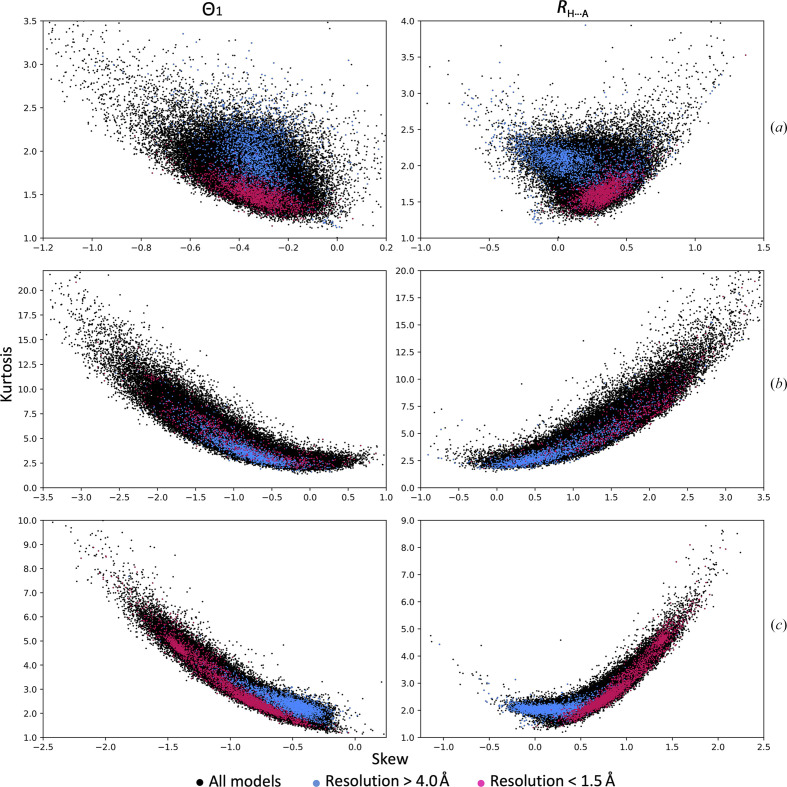
Distribution of skew versus kurtosis for Θ_1_ angles and *R*_H⋯A_ distances for all, high- and low-resolution models in the PDB shown for α-helices (*a*), β-sheets (*b*) and all (*c*) atoms.

**Figure 6:**
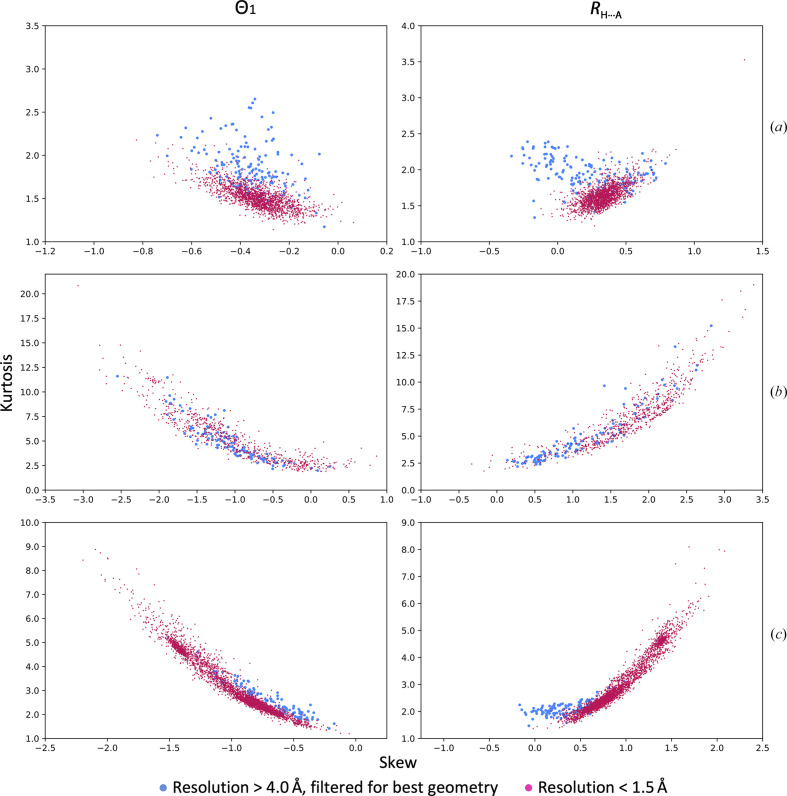
Distribution of skew versus kurtosis for Θ_1_ angles and *R*_H⋯A_ distances for all, high- and low-resolution models in the PDB shown for α-helices (*a*), β-sheets (*b*) and all (*c*) atoms. In contrast to Fig. 5 ▸, all PDB models are not shown and low-resolution models were filtered using the same geometrical criteria as applied to the high-resolution set (see Section 2 for details).

**Figure 7:**
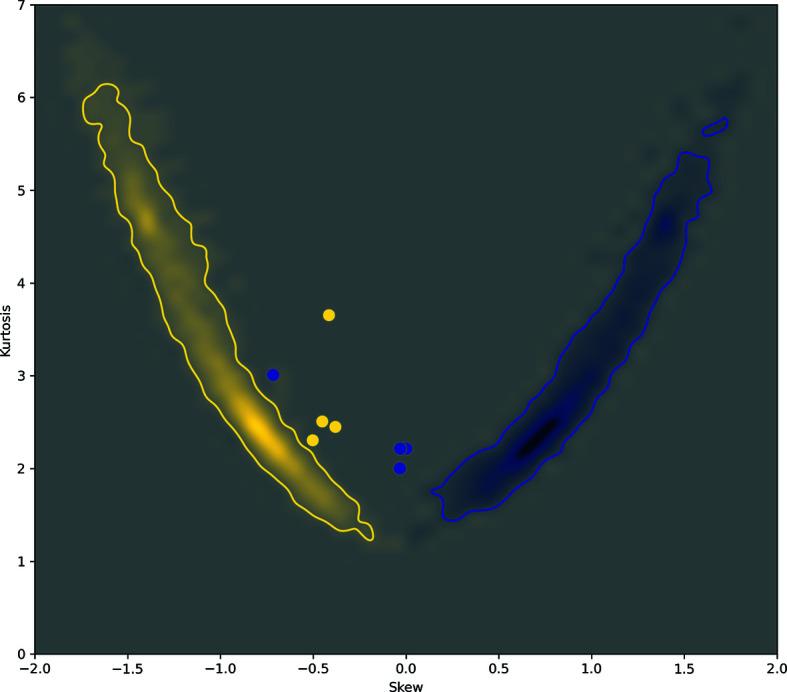
Skew and kurtosis of the Θ_1_ angle (yellow) and the *R*_H⋯A_ distance (blue) distributions for the models in Table 1 ▸ (PDB entries 5j1f, 5xb1, 6akf and 6mdo; values calculated for the entire models) shown with dots and overlaid with the skew and kurtosis obtained for all quality-filtered high-resolution models. The heat map qualitatively represents the distribution of the skew and kurtosis from Fig. 5 ▸(*c*) as a probability distribution; more saturated colors represent a higher probability of having a particular pair of skew and kurtosis values. The contours (solid lines) encompass the contiguous regions of probability values on the plot that were calculated using a statistically significant number of data points.

**Figure 8:**
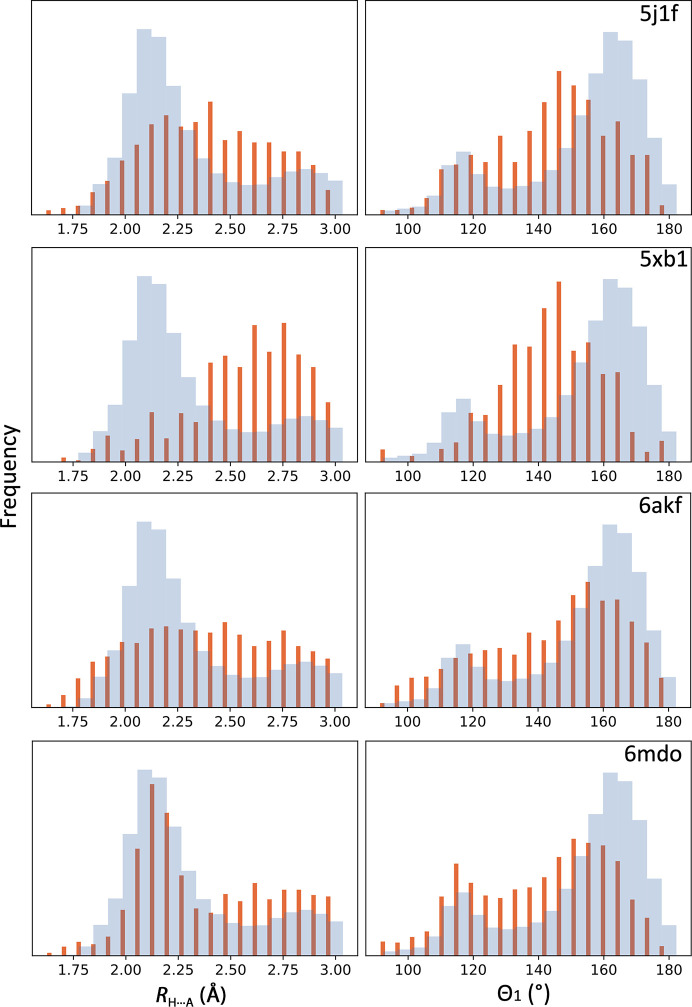
Histograms of *R*_H⋯A_ distances and Θ_1_ angles for the four models in Table 1 ▸ (PDB entries 5j1f, 5xb1, 6akf and 6mdo; red bars) overlaid with the distribution of these values derived from all quality-filtered high-resolution (1.5 Å and better) models from the PDB.

Fig. 5 ▸ shows a similar scatter plot of skew versus kurtosis but now considering all, low- and high-resolution models from the PDB. Notably, the distributions of low- and high-resolution models are rather well separated. As we pointed out earlier, possible reasons why these distributions vary between low- and high-resolution models are the inability of low-resolution data to capture and maintain the geometric features of secondary structure and simplistic modeling tools, which lead to less realistic atomic models (as these distributions reveal). We expect that models with skew and kurtosis values far from the values obtained for high-quality structures indicate model deficiencies. Filtering the low-resolution subset of models using the same geometrical criteria as we applied to high-resolution models still leaves a substantial number of models (Fig. 6 ▸). These remaining low-resolution models possess the same overall validation metrics as the high-resolution set, yet their hydrogen-bond parameters vary quite significantly. This suggests that these models still have oddities that were not flagged by standard validation criteria. For example, showing the skew and kurtosis of the hydrogen-bond parameters for models from Table 1 ▸ with respect to the reference distribution indicates that these models may have unlikely geometries (Fig. 7 ▸). Indeed, the histogram of Θ_1_ and *R*_H⋯A_ values for these models differ significantly from those observed for high-resolution models. A model with a more realistic geometry would have Θ_1_ and *R*_H⋯A_ values that follow the expected distributions (Fig. 8 ▸).

**Figure 9:**
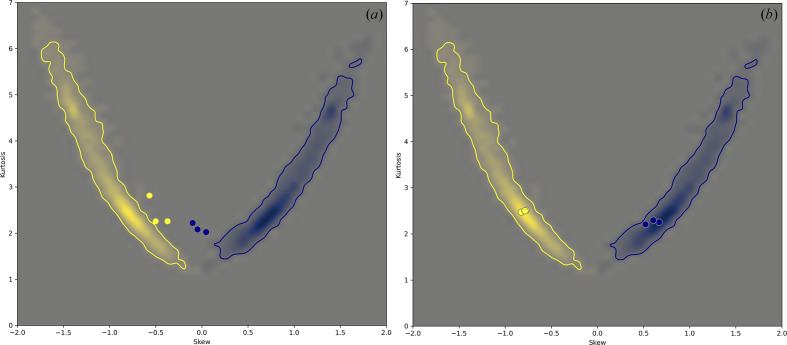
Overall kurtosis and skewness of the Θ_1_ angle (yellow) and *R*_H⋯A_ distance (blue) distributions for selected low-resolution models (*a*) compared with 100% homologous structures at high resolution (*b*). The low-resolution models were PDB entries 1jkt (3.5 Å; Tereshko *et al.*, 2001 ▸), 1z8l (3.5 Å; Davis *et al.*, 2005 ▸) and 4yj3 (3.8 Å; McNamara *et al.*, 2015 ▸) and the corresponding high-resolution models were PDB entries 4pf4 (1.1 Å; K. Temmerman, B. Simon & M. Wilmanns, unpublished work), 5o5t (1.4 Å; C. Barinka, Z. Novakova & L. Motlova, unpublished work) and 5iyz (1.8 Å; Waight *et al.*, 2016 ▸). See Fig. 7 ▸ for the definition of the heat maps.

To validate the method further, we compared the distribution of Θ_1_ and *R*_H⋯A_ parameters in selected low-resolution models that have higher resolution homologues. Three low-resolution models, PDB entries 1jkt (3.5 Å), 1z8l (3.5 Å) and 4yj3 (3.8 Å), have 100% sequence-identical high-resolution homologues, 4pf4 (1.1 Å), 5o5t (1.4 Å) and 5iyz (1.8 Å), respectively, that differ from each other by a root-mean-square deviation of less than 1 Å calculated over main-chain atoms. Now checking the overall skew and kurtosis values of Θ_1_ and *R*_H⋯A_ distributions for these models, we observe that the values for the high-resolution models considered here belong to the expected regions, while this is not the case for the low-resolution models (Fig. 9 ▸). We emphasize, however, that as with most statistics-based validation criteria, an *outlier* does not necessarily equate to *wrong* or *incorrect*; instead, it is meant to raise a warning and prompt additional checks.

**Figure 10:**
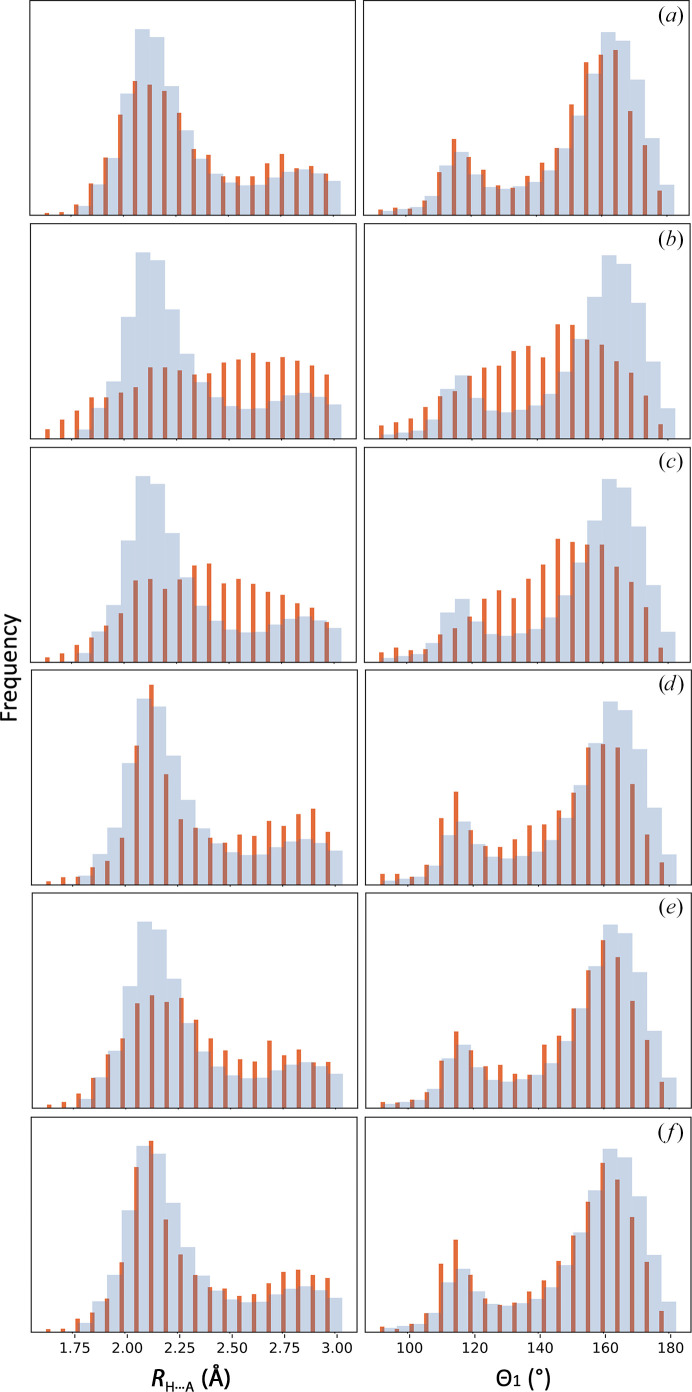
Distribution of the hydrogen-bond parameters for PDB entry 5iyz (red bars) overlaid with the distribution of values derived from all quality-filtered high-resolution (1.5 Å and better) models from the PDB: (*a*) the original model as deposited in the PDB, (*b*) the model perturbed with *phenix.dynamics* and the models after refinement using (*c*) only standard restraints, (*d*) standard restraints with the addition of secondary-structure and Ramachandran plot restraints, (*e*) standard restraints plus reference-model restraints and (*f*) standard, secondary-structure, Ramachandran plot and reference-model restraints.

Finally, to illustrate how the choice of refinement strategy can affect the proposed validation metric, we conducted the following numeric experiment using the model of the tubulin–MMAE complex (PDB entry 5iyz) that was originally refined against relatively high (1.8 Å) resolution data. The model has a distribution of hydrogen-bond parameters that matches the expected distributions (Figs. 10 ▸ *a* and 11 ▸ *a*). We then perturbed this model by introducing an r.m.s.d. of 0.7 Å into the co­ordinates using molecular dynamics (*phenix.dynamics*); this amount of perturbation is larger than a typical positional error estimate for well refined crystal structures, yet is within the convergence radius of refinement. Next, to mimic the low-resolution refinement scenario, we refined the perturbed model against the original data truncated at 4 Å resolution using *phenix.refine* (Afonine *et al.*, 2012 ▸). For the refinement, we considered the following four strategies for choices of model-geometry restraints: (i) using only empirical restraints on bond lengths, bond angles, dihedral angles, chiralities, planarities and repulsion (*standard restraints*;^1^ Engh & Huber, 1991 ▸; Grosse-Kunstleve *et al.*, 2004 ▸), (ii) using standard restraints with the addition of secondary-structure and Ramachandran plot restraints, (iii) using standard restraints plus the reference-model restraints, with the reference being the original unperturbed model, and (iv) using standard restraints together with secondary-structure, Ramachandran plot and reference-model restraints.

The perturbed model deviates quite notably from the reference distribution (Figs. 10 ▸ *b* and 11 ▸ *b*). This can be traced to the use of only standard restraints in *phenix.dynamics*. Using only standard restraints in the low-resolution refinement (Figs. 10 ▸ *c* and 11 ▸ *c*) also results in distorted distributions. It is clear that the low-resolution data do not contain sufficient information to resolve and maintain the hydrogen-bonding network. Supplementing the standard set of restraints with additional *a priori* known information about the model, such as secondary structure and the distribution of main-chain torsion angles (Ramachandran plot), or using the high-resolution information about the model (as reference-model restraints) can substantially improve the distribution of hydrogen-bond parameters, yet it does not make them match the distribution from the original high-resolution model (Figs. 10 ▸ *c*–10 ▸ *f* and 11 ▸ *c*–11 ▸ *f*). In all cases the refinement converged to the initial unperturbed model within 0.3 Å r.m.s.d., which is within the various estimates of coordinate error for refined models reported in the literature [see, for example, Rupp (2009 ▸) and references therein].

## 4. Conclusions

Using geometric restraints in low-resolution refinement that are usually used for the validation of atomic models, such as the Ramachandran plot, can diminish the validating power of these tools. New validation metrics are desirable to bootstrap existing validation methods. Here, we introduced a new validation tool that is based on analysis of the hydrogen-bond parameter distribution of a quality-filtered subset of high-resolution PDB models. These distributions can be characterized by skewness and kurtosis, and they appear to have a very narrow and specific shape that can be tabulated and used as a reference with which to compare new structures (similarly to Ramachandran plot or rotamer side-chain distributions). We used a set of selected models to demonstrate the efficacy of the method. We recommend a qualitative interpretation of the results obtained using this new validation method: a model that does not match the tabulated distributions of hydrogen-bond parameters is not necessarily wrong, but rather deserves a closer inspection in order to explain why it does not follow the expected distributions. The method has been implemented in *cctbx* and *Phenix*.

## Figures and Tables

**Figure 11:**
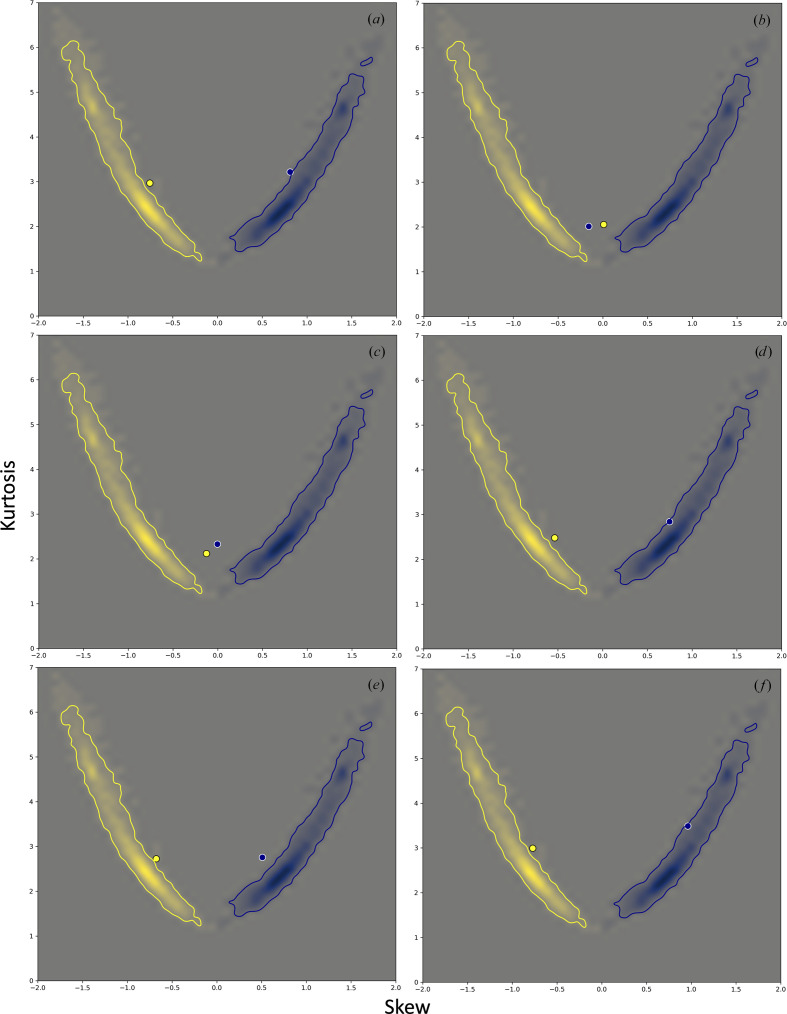
Skew and kurtosis of the Θ_1_ angle (yellow) and *R*_H⋯A_ distance (blue) distributions for the same models as reported in Fig. 10 ▸.

**Table 1:** Examples of models with nearly perfect overall model-geometry statistics but an unlikely distribution of residues in the Ramachandran plot: PDB entries 5j1f (Ortega *et al.*, 2016 ▸), 5xb1 (Ahn *et al.*, 2018 ▸), 6akf (Nakamura *et al.*, 2019 ▸) and 6mdo (White *et al.*, 2018 ▸)

PDB code	5j1f	5xb1	6akf	6mdo
Resolution (Å)	3	4	8	3.9
R.m.s.d.s				
Bond lengths (Å)	0.004	0.007	0.011	0.014
Angles (°)	0.86	0.7	1.59	1.32
Ramachandran plot				
Favored (%)	99.5	98.0	98.2	99.7
Outliers (%)	0	0	0	0
Rotamer outliers (%)	0	0	0	0
Clashscore	0	4	8	7
C^β^ deviation (%)	0	0	0	0

## Footnotes

1 Most crystallographic model-refinement packages use this set of geometric restraints in one form or another as the default choice.
